# Prevalence and severity of depression among Iraqi patients with systemic lupus erythematosus: A descriptive study

**DOI:** 10.31138/mjr.28.3.142

**Published:** 2017-09-29

**Authors:** Zahraa Adnan Abd-Alrasool, Faiq I. Gorial, Mushtaq T. Hashim

**Affiliations:** Rheumatology Unit, Baghdad Teaching Hospital, Baghdad, Iraq,; 2 Department of Medicine, College of Medicine, University of Baghdad, Baghdad, Iraq

**Keywords:** Depression, SLE, DSM5, SLEDA

## Abstract

**BACKGROUND:**

Systemic lupus erythematosus (SLE) patients have high risk for depression which is a potentially life-threatening disorder.

**OBJECTIVES:**

To evaluate the prevalence and severity of depression in a sample of Iraqi patients with SLE if present.

**PATIENTS AND METHODS:**

This cross-sectional study involved 60 patients with SLE diagnosed according to revised American College of Rheumatology (ACR) classification criteria. Demographics and clinical data were collected. All patients were screened for depression by using the diagnostic and statistical manual of mental disorders-5 (DSM5) diagnostic criteria of depression. Severity of their depression was determined by using the Beck Depression Inventory criteria.

**RESULTS:**

The prevalence of depression was 31.7%. A severe form of depression was observed in 13.3% of SLE cases, moderate depression in 10%, and a mild degree of depression was 8.3% of the cases. Patients with high SLE disease activity index (SLEDAI score >12) had an obviously higher rate of depression (40%) compared to 20% among those with mild or moderate disease. There was no important or statistically significant difference in median SLEDAI score between depression severity categories (p > 0.05).

**CONCLUSIONS:**

Prevalence of depression in SLE patients was relatively high. SLE disease activity increase depression rate.

## INTRODUCTION

Systemic lupus erythematosus (SLE) is a prototypic multisystem autoimmune disorder with a broad spectrum of clinical presentations encompassing almost all organs and tissues.^1^ In patients with SLE experience, there is a loss of self-tolerance result with abnormal immunological function and the production of autoantibodies, which lead to the formation of immune complexes that may adversely affect healthy tissue.^2^

Neuro-psychiatric symptoms are frequent in SLE with a range between 13% to 75%. The psychiatric symptoms may vary from mild personality disorders to severe psychotic behavior.^3^ Depression is a debilitating comorbidity common among patients with (SLE). Studies cited a broad range of prevalence rates of depression ranging from 17–75% and recent estimates suggest that point prevalence rates of clinically significant symptoms and lifetime incidence of Major Depressive Disorder occur in approximately half of patients with SLE.^4^

Depression has substantial impact on quality of life, disease outcomes and health care costs. Tumor necrosis factor alpha (TNF-α) is deeply related to pathogenesis of neurodevelopmental disorders, especially depression.^5^ Although pathways are not fully unraveled, immune and disease-related factors have been found to be associated with depression in SLE. In the general population, racial minorities, women and people from lower socioeconomic status have increased risk. Thus, SLE patients of minority groups and SLE women carry additional factors to those directly associated with the disease.^3^

Due to the potentially debilitating nature of the disease and relatively early onset for many women, SLE can pose multiple challenges and disrupt life goals throughout adulthood. Previous studies have found higher levels of psychiatric disturbance in patients with SLE, particularly depression or distress.^6^

Quickly identifying patients with sufficient depressive symptoms requiring treatment or referral to specialty care is an ongoing challenge in the clinic, particularly with overlapping somatic symptoms common to both SLE and depression.^7^ The management of depression in lupus rests on a combination of treating the underlying lupus itself as well as adding antidepressant therapy.^8^

The high prevalence of depression in patients with (SLE) may result from the psychosocial impact of this chronic disease as well as from a lesion of the central nervous system (CNS). Published evidence points to the participation of biochemical and neurophysiological changes, induced by cytokines, in the development of neuropsychiatric symptoms through activation of the enzyme indoleamine 2,3-dioxygenase (IDO).^9^

The alteration of neurotransmitters’ bioavailability, the modification of neuroplasticity and neurogenesis and the overstimulation of certain neural circuits and cytokines are capable of causing mood swings and depression. On the other hand, the Hypothalamic-Pituitary-Adrenal (HPA) axis dysfunction correlates with neurophysiological changes involved in depression.^9^ Moreover, cerebro-reactive autoantibodies present in the cerebrospinal fluid (CSF), such as anti-N-methyl-D-aspartate (NMDA) and anti-ribosomal P can cause significant damage to neurons in brain areas which are relevant to humor and behavior, potentially leading to depressive symptoms.^9^ The aim of the study was to evaluate the prevalence and severity of depression in patients with SLE.

## PATIENTS AND METHODS

### Study design

This cross-sectional study was conducted at the Rheumatology Unit Department of Medicine in Baghdad Teaching Hospital from September 2015 to May 2016. Ethical approval was taken from medical department in Baghdad teaching hospital and college of medicine. Informed consent was taken from the individuals for admission in the study.

### Sample selection

A total of 60 of Iraqi patients with SLE aged above 18 years were enrolled in the study, All SLE patients included in the study met the revised American College of Rheumatology (ACR) classification criteria.^10^ Patients were excluded if they had another overlapping inflammatory or connective tissue disease or another chronic illness.

### Data collection

Sociodemographic data and medications were recorded. Disease activity for SLE was assessed with the SLE Disease Activity Index (SLEDAI).^11^ Case histories and personal information were assessed: age, gender, SLE disease duration, body mass index (BMI), smoking status marital status unemployment, educational status, crowding index, and history of central nervous system (CNS) involvement. All subjects were screened for depression by using the DSM5 diagnostic criteria of depression.^12^ A semi-structure interview was done depending on DSM5 criteria for depressive disorders and then distributed to five experts for further evaluation, translation to Arabic and back translation was also done with correlation coefficients above 85 percent. Severity of their depression (mild, moderate, sever) was determined by using the Beck Depression Inventory criteria.^13^

### Laboratory measurement

Laboratory tests included full blood count, blood urea, serum creatinine, aspartate transaminase (AST), alanine transaminase (ALT), urinalysis, antinuclear antibodies, anti-double stranded-DNA (anti-ds-DNA) antibodies, complements components, and ribosomal p antibodies.

### Statistical analysis

Statistical analyses were done using SPSS version 23. Kolmogorov-Smirnov test was used to assess the normality of distribution of quantitative variables. Ages were normally distributed and presented as mean ± SD. SLEDAI score was shown to be non-normally distributed quantitative variables. Categorical variables were represented as numberσ (percentages). Correlation between disease activity and depression was assessed by spearman correlation. P value less than 0.05 was considered statistically significant.

## RESULTS

The age of SLE patients ranged between 13 to 60 years (mean, 33.6 ± 11[SD] years). Those younger than 25 years represented 23.3%, while those 45 years and older constituted 21.7%. The majority of sample was female (96.7%). Obese subjects constituted 28.3% of the total sample, while those with normal BMI represented 36.7% of the group. Other baseline characteristics of the patients are shown in **Table 1**.

**Table 1:** Demographic and clinical characteristics of 60 SLE patients

**Variables**	**N**	**%**

Age group (years)		
<25	14	23.3
25–44	33	55.0
45+	13	21.7
Female	58	96.7
BMI categories (Kg/m2)		
Normal (<25)	22	36.7
Overweight (25–29.9)	21	35.0
Obese 30+	17	28.3
Marital status		
Single	19	31.7
Married	41	68.3
Employment		
Employed	55	91.7
Unemployed	5	8.3
Educational level		
No formal education (illiterate / read & write)	7	11.7
primary school	23	38.3
secondary school	17	28.3
college/higher education	13	21.7
Crowding index		
<2	13	21.7
(2–3)	31	51.7
4+	16	26.7
CNS involvement Positive	12	20.0
Antinuclear antibodies Positive	40	87.0
Anti double stranded DNA Positive	27	62.8
Decrease C3 C4 Positive	14	36.8
SLE Disease duration (years)-categories		
<2	11	18.3
(2–9)	39	65.0
10+	10	16.7
Disease activity		
Mild/moderate	25	41.7
Severe disease	35	58.3
History of drugs used		
Prednisolone	48	80.0
Hydroxychloroquine	33	55.0
cyclophosphamide	19	31.7
Azathioprine	16	26.7
Mycophenylate	9	15.0
MTX	2	3.3
Chloroquine	1	1.7

**Figure 1:**
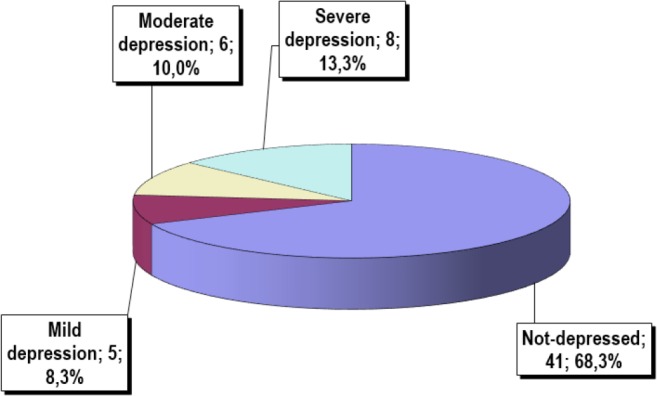
Pie chart showing the relative frequency of selected severity categories of diagnosed depression in the study sample.

As shown in **Table 2**, around a third of cases (31.7%) were found to have evidence of depression. It is expected that the prevalence of depression among SLE population may range between as low as 20.3% to as high as 45% with a confidence level of 95%. A severe form of depression was observed in 13.3% of examined SLE cases, moderate depression was 10% while a mild degree of depression was elicited in 8.3% of the case series, **Figure 1**.

**Table 2:** The prevalence of depression in the study sample.

	**N**	**%**	**95% confidence interval for prevalence rate**
Depression	19/60	31.7	(20.3% – 45.0%)

Furthermore, cases with a high disease activity disease evaluated by SLEDAI score >12 had an obviously higher rate of depression (40%) compared to 20% among those with mild or moderate form of SLE disease activity. The association between disease activity and depression, however, failed to reach the level of statistical significance.

## DISCUSSION

There are several reports available concerning prevalence of depression in the course of systemic lupus erythematosus. To the best of our knowledge, there were no previous reports about prevalence of depression among Iraqi patients with systemic lupus erythematosus. The purpose of this study was to evaluate the prevalence of depression in Iraqi patients with SLE.

This study revealed that around a third of cases (31.7%) were found to have evidence of depression. This finding was close to the results of Drenkard et al. study^6^ which reported depression prevalence was (31%) and Adeli et al. study^14^ which reported that depression prevalence in SLE was 33.3%. In addition, this finding was comparable with Meszaros et al. study^15^ in which depression was present in in up to 39% of patients, and the authors suggested that genetic and environmental factors (e.g., ultraviolet light, retroviruses, and medications) and patient’s reaction to the illness may play a role in the pathogenesis.

The high prevalence of depression in patients with SLE may result also from the psychosocial impact of this chronic disease as well as from a lesion of the central nervous system (CNS), and the participation of biochemical and neurophysiological changes induced by cytokines.^9^ The alteration of neurotransmitters’ bioavailability, the modification of neuroplasticity and neurogenesis and the overstimulation of certain neural circuits and cytokines, also Hypothalamic-Pituitary-Adrenal (HPA) axis dysfunction correlates with neurophysiological changes involved in depression. Furthermore, autoantibodies present in the cerebrospinal fluid (CSF), such as anti-N-methyl-D-aspartate (NMDA) and anti-ribosomal P can cause significant damage to neurons in brain areas, potentially leading to depressive symptoms.^9^

In Iraq depression have been reported in few previous studies. Al-Hamzawi et al.^16^ observed that Major Depression Episode is a commonly occurring disorder in the Iraqi general population and is associated with considerable disability and low treatment. Hasan et al.^17^ demonstrated that depression is very common among Parkinson’s disease patients; however, no specific type of depression could be identified in those patients. There was significant association between depression and autonomic involvement, insomnia and dysphagia in Parkinson’s disease. Depression in Parkinson’s disease was not found to be related to the age of onset or to the duration of the disease. Hamody et al.^18^ reported that Depression is common in this group of Iraqi hemodialysis patients and its prevalence is comparable to the results of similar studies in other societies.

In the current study the severe form of SLE was observed in 58.3% of our patients. In contrast to Shakeri et al. study^19^ who used BID to assess depression severity and found that the prevalence of severe depression was estimated to be about 20%. Possible explanation of this difference may be related to difference in sample size and data measurement.

A limitation of this study is small sample size due to short period of the study and rarity of the disease, however this is the first study in Iraq up to our knowledge that assessed depression in SLE. With strict inclusion and exclusion criteria.

In conclusion, the prevalence of depression among SLE patients was relatively high (31.7%). Prevalence of depression increase with the increase in disease activity. Early and frequent depression screening for patients with SLE to early diagnose and treat the depression and to prevent complications and larger sample size and longer disease duration study to further validate the findings of this study are suggested.
